# N-butyl cyanoacrylate glue: the most misunderstood hero in interventional radiology

**DOI:** 10.1186/s42155-026-00653-3

**Published:** 2026-02-14

**Authors:** Romaric Loffroy

**Affiliations:** Department of Vascular and Interventional Radiology, Image-Guided Therapy Center, François-Mitterrand University Hospital, 14 Rue Paul Gaffarel, BP 77908, Dijon, 21079 France

In the evolving landscape of interventional radiology, the range of embolic agents available to contemporary practice has expanded considerably. Calibrated microspheres, detachable coils, and a variety of liquid embolic agents now offer multiple effective options tailored to specific clinical scenarios [1]. Embolic agent selection is inherently context-dependent, influenced by factors such as vascular anatomy, procedural objectives, and operator experience. Within this diverse armamentarium, n-butyl cyanoacrylate (NBCA) has maintained a distinct and enduring role. Its technical versatility and well-established clinical performance support its continued relevance across a broad spectrum of embolization procedures [2].

## A technically superior tool: safe, fast, visible, versatile

NBCA is often perceived as a demanding agent—a liquid glue that polymerizes instantly upon contact with ionic fluids such as blood, requiring a practiced hand and thoughtful technique. But when mastered, its very properties make it the most technically elegant and efficient embolic material available [1, 2].

## Safety through control and predictability

Concerns regarding unintended migration have historically influenced perceptions of NBCA use. However, when appropriately prepared and deployed, NBCA can offer a high degree of procedural control, particularly in experienced hands. Admixture with ethiodized oil allows modulation of polymerization kinetics, enabling the operator to tailor the depth of penetration to specific procedural goals. This level of control may be more difficult to achieve with particulate embolics in selected settings [3]. As its mechanism of action does not depend on blood flow stasis, NBCA can be especially advantageous in high-flow lesions, including arteriovenous malformations (AVMs) and traumatic pseudoaneurysms, where coils or particles may be associated with an increased risk of incomplete occlusion. Institutional familiarity and operator experience remain important determinants of safe and effective use [4, 5].

## Speed and efficiency

NBCA is associated with rapid embolization and can offer meaningful procedural efficiency in appropriately selected cases. Following injection, polymerization occurs quickly, often resulting in prompt vessel occlusion. In emergent clinical scenarios—such as massive hemoptysis, active gastrointestinal bleeding, or traumatic hemorrhage—timely hemostasis is critical. In these settings, the rapid occlusive properties of NBCA may facilitate faster stabilization compared with embolic agents that rely on progressive flow reduction or thrombosis [6, 7]. That said, procedural speed is influenced by multiple factors, including vascular anatomy, lesion characteristics, and embolic selection, and similar efficiency may be achieved with alternative agents in certain contexts. The absence of a prolonged waiting period between delivery and effect can nonetheless be advantageous when time is a limiting factor.

## Visibility and feedback

The radiopacity of NBCA when mixed with ethiodized oil provides real-time visual feedback during injection. The operator can watch the material advance under fluoroscopy, adjusting the injection rate or catheter position as needed. Unlike particles, which can be invisible beyond the catheter tip, NBCA is visible from the start to the end of its journey—a vital advantage in both safety and precision [8, 9].

## Versatility beyond comparison

NBCA is the true multitasker of embolic agents. It can be used through microcatheters or small diagnostic catheters, for arteries, veins, or lymphatics, and in both high- and low-flow systems [1, 2]. It can occlude a bleeding branch, seal an aneurysmal neck, close a shunt, or mark a surgical plane. Its adaptability makes it invaluable across neurovascular, peripheral, oncologic, and emergency applications [3, 7, 10].

## Durable occlusion and better outcomes as clinical strength

While technical performance is essential, clinical outcomes ultimately define the value of any embolic agent. In this regard, NBCA has demonstrated effective embolization and durable occlusion across several well-established clinical indications. Polymerization of NBCA results in the formation of a solid intravascular cast, creating a permanent mechanical barrier. Consequently, recanalization—more frequently observed with temporary or partially occlusive agents—appears to be uncommon in appropriately selected cases.

In established applications such as gastrointestinal bleeding, multiple studies have reported high primary technical success rates and reduced rebleeding with NBCA compared with particulate embolics or coils [3, 7]. Similarly, durable outcomes have been described in bronchial artery embolization, including in patients with hypertrophic or tortuous vessels or in the presence of systemic–pulmonary shunts [6, 11]. Favorable and sustained results have also been reported in venous interventions, including varicocele embolization and pelvic congestion syndrome, where the occlusive properties of NBCA may confer advantages over alternative embolic materials [12].

By contrast, the role of NBCA in newer applications such as prostatic artery embolization and bariatric embolization is still evolving. Early and emerging data suggest potential benefits of NBCA over particulate agents in selected patient populations; however, the current evidence base remains limited, and further prospective studies are required to define its comparative effectiveness and long-term outcomes in these settings [8, 9, 13].

## Performance in complex vascular territories

In neuro-interventions, NBCA remains a benchmark for treating brain AVMs and dural arteriovenous fistulas [10, 14]. Despite the advent of newer liquid embolics, glue offers unmatched penetration and long-term occlusion when used judiciously [14]. In oncology, its role in preoperative devascularization of hypervascular tumors or as part of combination therapies continues to demonstrate robust outcomes, reducing intraoperative blood loss and improving surgical field conditions [15].

## Cost-effectiveness and worldwide availability

NBCA also distinguishes itself by being remarkably cost-effective and universally accessible. A single vial can serve multiple patients, requires no proprietary delivery system, and has a long shelf life. Unlike newer liquid embolics that demand expensive catheters or dedicated micro-systems, NBCA can be delivered with standard equipment found in nearly every angiography unit [2]. Its affordability and reliability have made it the embolic of choice in many regions where healthcare resources are constrained, ensuring that even complex interventions can be performed safely without financial or logistical barriers. In this sense, NBCA is not only technically versatile but also globally democratic, embodying the essence of practical, life-saving medicine.

## Mastery over myth

Hesitation surrounding NBCA use is often related to its perceived learning curve and the technical demands associated with rapid polymerization. NBCA requires careful preparation and precise execution; however, with appropriate training, adherence to standardized techniques—such as continuous injection, catheter flushing with dextrose solution, and thoughtful selection of dilution ratios—and increasing operator experience, its use can become predictable and well controlled.

Operator expertise and institutional familiarity are important determinants of safe and effective application. When used by appropriately trained interventionalists, NBCA remains a technically demanding yet reliable embolic agent within the contemporary embolization armamentarium [1, 2].

## Glue as the indispensable agent

In conclusion, if I could keep only one embolic agent in the lab, it would be NBCA. Not out of nostalgia, but because of what it represents: technical mastery, procedural efficiency, clinical reliability, and universal applicability. It is safe when used with respect, fast when seconds matter, visible when precision counts, and versatile when the unexpected arises. NBCA is not just another embolic—it is the embolic of last resort and first choice, the one that can perform in any environment, under any pressure, for any pathology. In a world enamored with complexity, it remains elegantly simple: a drop of glue that can save a life.

## Data Availability

Not applicable.
